# Effects of digital health interventions on anxiety, depression, and quality of life in colorectal cancer patients: a meta-analysis of randomized controlled trials

**DOI:** 10.3389/fonc.2026.1832924

**Published:** 2026-06-02

**Authors:** Mei-Ying Song, Min Li, Ling Tu, Jie Yang

**Affiliations:** 1 Colorectal Cancer Center, West China Hospital, Sichuan University, Chengdu, Sichuan, China; 2 Departmentof General Surgery, West China Hospital, Sichuan University, Chengdu, Sichuan, China; 3 Trauma Medical Center, West China Hospital, Sichuan University, Chengdu, Sichuan, China

**Keywords:** anxiety, colorectal cancer, depression, digital health interventions, meta-analysis, quality of life

## Abstract

**Background:**

Colorectal cancer (CRC) patients generally experience distressing psychological symptoms, including anxiety and depression, which significantly diminish their quality of life. Digital health interventions have emerged as a promising complementary approach to managing these challenges. This meta-analysis of randomized controlled trials aimed to determine the effects of digital health interventions on anxiety, depression, and quality of life in CRC patients.

**Methods:**

A comprehensive literature search was conducted across ten databases (PubMed, Web of Science, Scopus, Embase, Cochrane Library, CINAHL, CNKI, VIP, WanFang, and CBM) from inception to February 28, 2026. Randomized controlled trials evaluating the effects of digital health interventions on anxiety, depression, and quality of life among CRC patients were eligible for inclusion. The Cochrane Risk of Bias tool 2.0 (ROB 2.0) was employed to assess methodological quality, and the Grading of Recommendations, Assessment, Development and Evaluation (GRADE) framework was applied to grade the certainty of evidence for each outcome. A random-effects model was used for all quantitative syntheses.

**Results:**

A total of 15 randomized controlled trials comprising 2018 CRC patients were included. Pooled analyses revealed that digital health interventions were associated with a potential reduction in anxiety (SMD = -0.90, 95% CI: -1.59 to -0.22, I² = 93%, low certainty evidence) and depression (SMD = -0.93, 95% CI: -1.64 to -0.21, I² = 94%, low certainty evidence), and improvement in quality of life (SMD = 0.78, 95% CI: 0.19 to 1.36, I² = 96%, low certainty evidence) compared to control conditions.

**Conclusions:**

Our findings revealed that digital health interventions hold promise in reducing anxiety and depression and enhancing quality of life among CRC patients. Given the limitations posed by substantial heterogeneity and low evidence certainty, high-quality randomized controlled trials are required to establish more definitive conclusions.

## 1 Introduction

Colorectal cancer (CRC) constitutes a significant global public health challenge (1). As the third most prevalent malignant tumor and the second leading cause of cancer-related mortality, it resulted in approximately 1.9 million new cases and 930000 deaths in 2020, with the disease burden projected to intensify further over the next two decades (2, 3). Although the widespread availability of screening technologies and advances in treatment options have significantly improved patient survival rates, the diagnosis of cancer, along with invasive treatments such as surgery, chemotherapy, and radiation therapy, imposes immense physical and psychological stress on CRC patients (4–6). CRC patients not only endure the suffering of physical symptoms but also commonly contend with severe psychological challenges (7). Anxiety and depression are the most common psychological disturbances experienced by CRC patients (8). Epidemiological studies indicated that the prevalence rates of anxiety and depression among CRC patients are as high as 57% and 47.2%, respectively (9). Both substantially impair their quality of life and lead to reduced treatment adherence and poor clinical health outcomes (10). Therefore, the effective management of these adverse psychological symptoms and the improvement of quality of life in CRC patients have emerged as critical priorities in contemporary supportive care for cancer patients.

Although conventional psychological support, such as in-person counseling and cognitive behavior therapy, has proven effective in reducing anxiety and depression among cancer patients, its accessibility for CRC patients is generally limited (11, 12). There are several reasons for this, including the uneven distribution of psychological services and the long waiting times for appointments (13). In addition, patients’ postoperative weakness or mobility difficulties make regular visits challenging (14). Furthermore, individuals with a permanent stoma may avoid face-to-face interactions owing to stigma, such as concerns about altered body image or social embarrassment resulting from stoma leakage (4). These barriers hinder the delivery of traditional psychological support to this population. In recent years, the adoption of digital health interventions has increased substantially, with a growing number of randomized controlled trials evaluating their effectiveness across diverse cancer populations (15, 16). These interventions employ smartphone applications, web-based programs, remote consultations, virtual reality, and wearable devices to transcend time and space constraints. They provide patients with convenient, personalized, and cost-effective support, including psychological care, physical activity promotion, nutritional education, medication adherence, and symptom self-management (17, 18). According to theoretical frameworks such as the Technology Acceptance Model and Self-Efficacy Theory, digital tools may enhance patients’ perceived self-efficacy and reduce barriers to care by providing accessible, personalized, and real-time support (19, 20). In this context, digital health interventions have been recognized by the World Health Organization for their role in enhancing access to healthcare, positioning them as a promising tool to overcome the aforementioned barriers (18).

A growing literature of randomized controlled trials has examined the effectiveness of digital health interventions among CRC patients. However, the conclusions drawn from these studies remain inconsistent. Several empirical studies have demonstrated that digital interventions can significantly alleviate anxiety and depression while improving quality of life (19–21). In contrast, other researches have indicated that their effects may be limited or lack clinical significance (22, 23). The heterogeneity may derive from variations in the intervention content, duration, control group design, and outcome measurement tools employed. Despite digital health interventions being regarded as a valuable complement to conventional care models, there remains a lack of systematic evidence-based research specifically targeting CRC patients. To address this knowledge gap, a meta-analysis of randomized controlled trials was conducted to determine the effects of digital health interventions on anxiety, depression, and quality of life among CRC patients.

## 2 Materials and methods

This meta-analysis adhered to the Preferred Reporting Items for Systematic Reviews and Meta-Analyses (PRISMA) guidelines (24), and the protocol was registered with PROSPERO (CRD420261333102).

### 2.1 Search strategy

Literature searches were conducted in PubMed, Web of Science, Scopus, Embase, Cochrane Library, CINAHL, CNKI, VIP, WanFang, and CBM from inception to February 28, 2026, with a restriction to English and Chinese publications. The search strategy was structured around three conceptual blocks: colorectal cancer, digital health interventions, and randomized controlled trials (RCTs). Within each block, terms were combined using the OR operator, and the three blocks were then combined using the AND operator. Boolean operators were used to combine terms within and across blocks. For each database, we used both its controlled vocabulary (such as MeSH for PubMed, Emtree for Embase) and free-text keywords in titles/abstracts, adapting the search syntax accordingly. To identify RCTs, we applied a validated RCT filter, which combines controlled vocabulary with free-text terms. The terms “randomized clinical trial” and similar variants were used as search keywords within the study design block, not merely as inclusion criteria. The search terms included colorectal neoplasms, colorectal cancer, colon cancer, rectal cancer, digital health, telemedicine, eHealth, mHealth, website, app, phone, email, text message, social media, virtual reality, wearable devices, and randomized clinical trial. To ensure reproducibility, the full PubMed search string with field tags and filters was: (“colorectal neoplasms”[MeSH] OR “colorectal cancer”[Title/Abstract] OR “colon cancer”[Title/Abstract] OR “rectal cancer”[Title/Abstract]) AND (“digital health”[MeSH] OR “telemedicine”[MeSH] OR “eHealth”[Title/Abstract] OR “mHealth”[Title/Abstract] OR “website”[Title/Abstract] OR “app”[Title/Abstract] OR “phone”[Title/Abstract] OR “email”[Title/Abstract] OR “text message”[Title/Abstract] OR “social media”[Title/Abstract] OR “virtual reality”[Title/Abstract] OR “wearable devices”[Title/Abstract]) AND (“RCT”[Title/Abstract] OR “randomized clinical trial”[Title/Abstract] OR “randomized controlled trial”[Title/Abstract] OR “randomized trial”[Title/Abstract] OR “randomized controlled trial”[Title/Abstract] OR “randomised trial”[Title/Abstract]). In addition, we manually screened the reference lists of all included studies to identify additional potentially eligible publications. We contacted study authors when needed, but grey literature was not searched due to time and resource constraints. Full details of the search strategies are provided in **Supplementary Table 1**.

### 2.2 Study selection and data extraction

All studies were selected based on the PICOS criteria. The inclusion criteria were: (1) Population: adult patients with colorectal cancer (≥ 18 years); (2) Intervention: digital health technologies, including websites, app, social media, telephone, or other digital tools; (3) Comparison: usual care, wait-list, and conventional education; (4) Outcomes: at least one of anxiety, depression, or quality of life was assessed and reported; (5) Study design: randomized controlled trials. These studies were excluded if they: (1) were duplicate publications; (2) were conference abstracts, protocols, case reports, or reviews; (3) lacked sufficient data for meta-analysis; (4) were not published in English or Chinese.

Data extraction was performed independently by two reviewers using a standardized form. The standardized form was validated prior to use by pilot-testing on a random sample of five included studies, with refinements made based on team discussion. Any discrepancies identified during the extraction process were resolved by consulting a third reviewer to achieve a final consensus. The following information was collected: first author, publication year, country, sample size, mean age, therapeutic stage, experimental group characteristics (a brief introduction of the intervention, frequency, and duration), control group, and outcome measures. Due to the general lack of follow-up data, we extracted post-intervention values when available, and change-from-baseline scores for studies that reported only these values. For multi-arm studies, we selected the digital health intervention arm that best matched the primary objective and used the concurrent control group. No missing standard deviations were present in the included studies. Therefore, no imputation was required.

### 2.3 Risk of bias and certainty of evidence assessment

The risk of bias and certainty of evidence assessment were conducted independently by two reviewers, with any discrepancies addressed by consulting a third researcher. The risk of bias of the included articles was assessed using the Cochrane Risk of Bias Tool 2.0 (ROB 2.0) (25), which evaluates studies across five domains: (1) the randomization process; (2) deviations from intended interventions; (3) missing outcome data; (4) measurement of the outcome; (5) selection of the reported result. Each study was assigned a judgment of “low,” “some concerns,” or “high” risk of bias.

The certainty of evidence regarding the effects of digital health interventions on the outcomes of anxiety, depression, and quality of life in CRC patients was evaluated using the Grading of Recommendations, Assessment, Development, and Evaluation (GRADE) framework (26). Evidence levels were categorized as “high,” “moderate,” “low,” or “very low” based on limitations in five domains: risk of bias, inconsistency, indirectness, imprecision, and publication bias.

### 2.4 Data analysis

The effect size was calculated using Hedges’ g as the standardized mean difference (SMD) with 95% confidence interval (CI). Hedges’ g provides a correction for small-sample bias, which is particularly relevant when some studies have limited sample sizes (27). The mathematical expression for SMD is: $\text{SMD}=\frac{\text{Difference in mean outcome between groups}}{\text{Standard deviation of outcome among participants}}$ Heterogeneity in the included studies was quantified using the Cochran’s Q test and the I² statistic. Given that an I² value ≥ 50% indicates substantial heterogeneity, a random-effects model with DerSimonian-Laird method was adopted to estimate the overall effect sizes. In addition, we calculated the 95% prediction interval (PI) to estimate the range within which the true effect size of a future individual study would fall (28). Subgroup analyses were conducted to explore potential sources of heterogeneity. Two subgroup analyses were pre-specified in our protocol: therapeutic phase and intervention duration. Additional subgroup analyses on intervention format, outcome measurement tool, and risk of bias were performed as exploratory analyses given the limited prior evidence and to better understand potential effect modifiers. The robustness of the meta-analysis findings was tested using a leave-one-out sensitivity analysis. For outcomes with at least 10 studies, publication bias was assessed using funnel plots and Egger’s. For outcomes with fewer than 10 studies, formal tests for publication bias were not performed due to low statistical power. All statistical analyses were conducted with Stata version 17.0.

## 3 Results

### 3.1 Selection results and study characteristics

**Figure 1:**
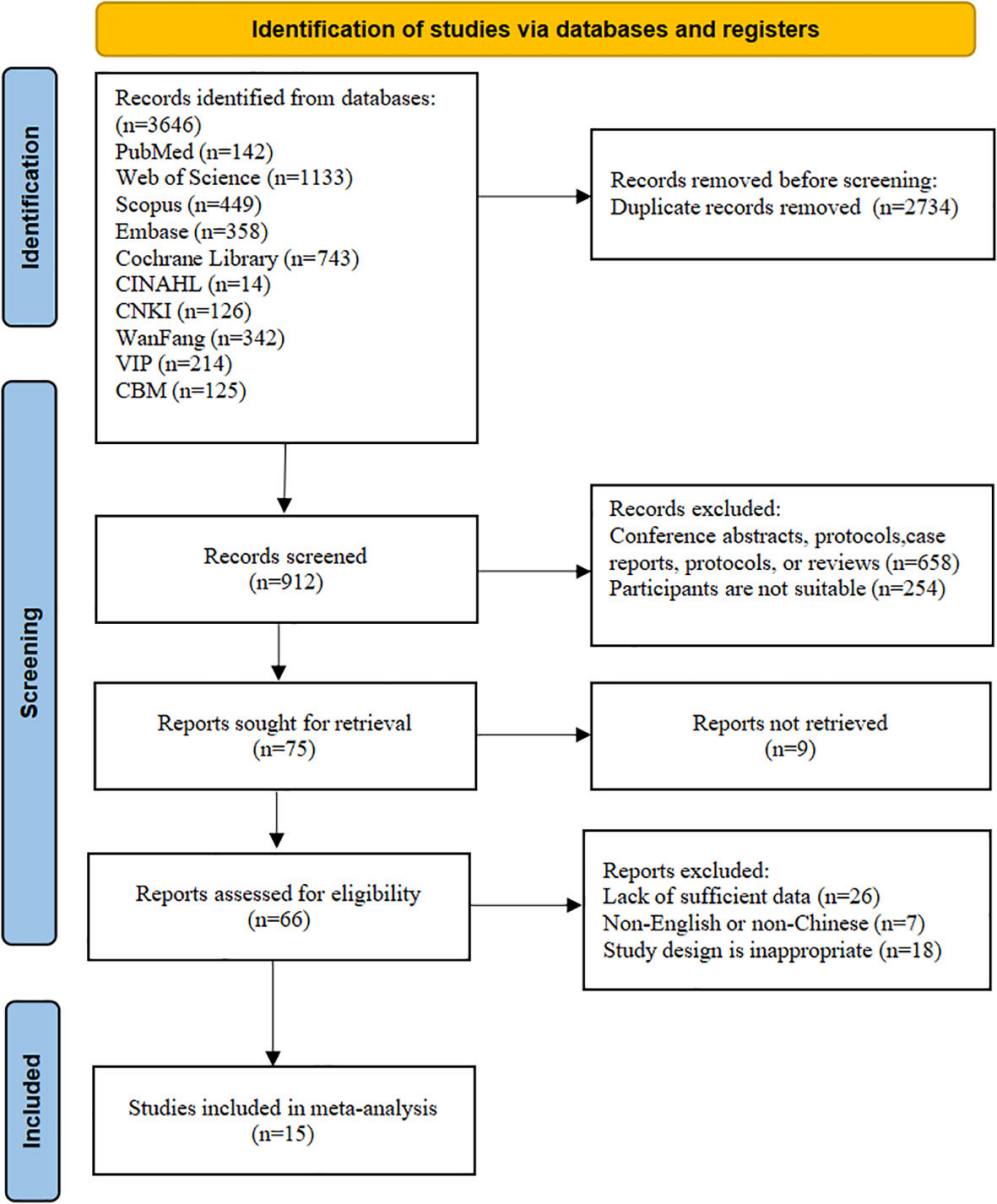
Flow diagram of study selection.

A total of 3646 records were retrieved from the database searches. After removing duplicates, 912 articles were screened by title and abstract. Of these, 66 met the preliminary inclusion criteria and were reviewed in full text, ultimately yielding 15 studies that met all eligibility criteria for inclusion in the meta-analysis. The study selection process is detailed in **Figure 1**. A total of 2018 CRC patients were enrolled across the 15 studies, which were published between 2014 and 2025. 15 trials were conducted in China (n = 6), the USA (n = 2), Australia (n = 2), Korea (n = 1), the Netherlands (n = 1), Canada (n = 1), Spain (n = 1), and Germany (n = 1), with sample sizes ranging from 31 to 756. Moreover, most participants were in the active treatment phase. Digital health interventions were delivered via telephone, apps, social media, websites, wearable devices, and virtual reality. The characteristics of the included studies are provided in **Table 1**.

**Table 1:** Characteristics of included studies.

Study	Country	Participants			Experimental group			Control group	Outcomes (instrument)
		Sample size	Mean age (years)	Therapeutic stage	Brief introduction	Format	Duration		
Chan et al., 2022 (29)	USA	EG: 20 CG: 21	EG: 55.6 ± 12.3 CG: 54.4 ± 10.6	Survivors	A pedometer and daily text message-based physical activity intervention program.	Telephone and wearable devices	12 weeks	Conventional education	Quality of life (FACT-C)
Custers et al., 2024 (19)	Netherlands	EG: 41 CG: 43	EG: 62.5 ± 9.5 CG: 64.9 ± 9.1	Survivors	A telephone- and web-based cognitive behavioral intervention program.	Website and telephone	14 weeks	Usual care	1. Anxiety (HADS-A) 2. Depression (HADS-D) 3. Quality of life (EORTC-QLQ-C30)
Dong et al., 2019 (20)	China	EG: 45 CG: 45	NA	Active treatment	A telephone-based reminiscence therapy program.	Telephone	6 weeks	Usual care	1. Anxiety (SAS) 2. Depression (SDS)
Hawkes et al., 2014 (21)	Australia	EG: 205 CG: 205	NA	Survivors	A telephone- and wearable device-based health coaching program.	Telephone and wearable devices	12 months	Usual care	Quality of life (FACT-C)
Hwang et al., 2025 (30)	Korea	EG: 17 CG: 17	EG: 62.18 ± 7.18 CG: 63.29 ± 7.10	Active treatment	An app-delivered physical activity intervention program.	APP	6 weeks	Conventional education	Quality of life (EORTC-QLQ-C30)
Kelleher et al., 2021 (22)	USA	EG: 14 CG: 17	59.5 ± 10.5	Survivors	A telephone-based coping skills training program.	Telephone	5 weeks	Usual care	Quality of life (FACT-G)
Moon et al., 2025 (23)	Canada	EG: 45 CG: 46	EG: 63.2 ± 13.0 CG: 65.2 ± 9.9	Active treatment	An app-based peer support and health education program.	App	6 months	Usual care	1. Anxiety (HADS-A) 2. Depression (HADS-D) 3. Quality of life (EORTC-QLQ-C30)
Rao and Chen. 2023 (31)	China	EG: 41 CG: 40	EG: 59.21 ± 9.16 CG: 58.72 ± 8.52	Survivors	A WeChat-based transitional care rehabilitation program for colorectal cancer patients with an ostomy.	Social media	3 months	Usual care	1. Anxiety (SAS) 2. Depression (SDS)
Rocamora GonzálezLin et al., 2022 (32)	Spain	EG: 39 CG: 43	EG: 63.7 CG: 66.4	Active treatment	An app-based mindfulness-based stress reduction program.	App	1 month	Usual care	1. Anxiety (HADS-A) 2. Depression (HADS-D) 3. Quality of life (WHOQOL-BREF)
Schrempf et al., 2023 (33)	Germany	EG: 31 CG: 31	EG: 60.4 ± 9.5 CG: 60.9 ± 9.7	Active treatment	A virtual reality fitness game to promote recovery in patients undergoing colorectal cancer surgery.	Virtual reality	1 month	Usual care	Quality of life (EQ-5D)
Wang et al., 2022 (34)	China	EG: 28 CG: 28	EG: 69.07 ± 6.53 CG: 67.50 ± 9.34	Survivors	A WeChat-based tailored nutritional intervention to provide personalized recommendations.	Social media	6 months	Usual care	Quality of life (EORTC-QLQ-C30)
Wang et al., 2023 (35)	China	EG: 25 CG: 25	NA	Survivors	A web-based dietary intervention with text messages.	Website and text messages	12 weeks	Waitlist	Quality of life (EORTC-QLQ-C30)
Young et al., 2014 (36)	Australia	EG: 387 CG: 369	EG: 68.6 ± 12.2 CG:67.0 ± 12.1	Active treatment	A centralized nurse-led telephone-based care coordination program.	Telephone	6 months	Usual care	Quality of life (FACT-C)
Yue et al., 2014 (37)	China	EG: 40 CG: 40	EG: 64.3 ± 6.5 CG:64.5 ± 10.3	Active treatment	A rehabilitation program combining an exercise intervention with virtual reality relaxation training, wearable devices, and social media	Virtual reality, wearable devices, and social media	1 month	Usual care	1. Anxiety (HADS-A) 2. Depression (HADS-D)
Zhang and Lin. 2024 (38)	China	EG: 35 CG: 35	NA	Active treatment	A WeChat-based group mindfulness-based stress reduction intervention.	Social media	2 months	Usual care	1. Anxiety (SAS) 2. Depression (SDS)

EG, experimental group, CG, control group, EORTC QLQ-C30, European Organisation for Research and Treatment of Cancer Quality of Life Questionnaire Core 30, EQ-5D, EuroQol Five-Dimensional Questionnaire, FACT-C, Functional Assessment of Cancer Therapy-Colorectal, FACT-G, Functional Assessment of Cancer Therapy-General, HADS-A, Hospital Anxiety and Depression Scale-Anxiety, HADS-D, Hospital Anxiety and Depression Scale-Depression, SAS, Self-Rating Anxiety Scale, SDS, Self-Rating Depression Scale, WHOQOL-BREF, World Health Organization Quality of Life Instrument-Brief Version.

### 3.2 Risk of bias assessment

**Figure 2:**
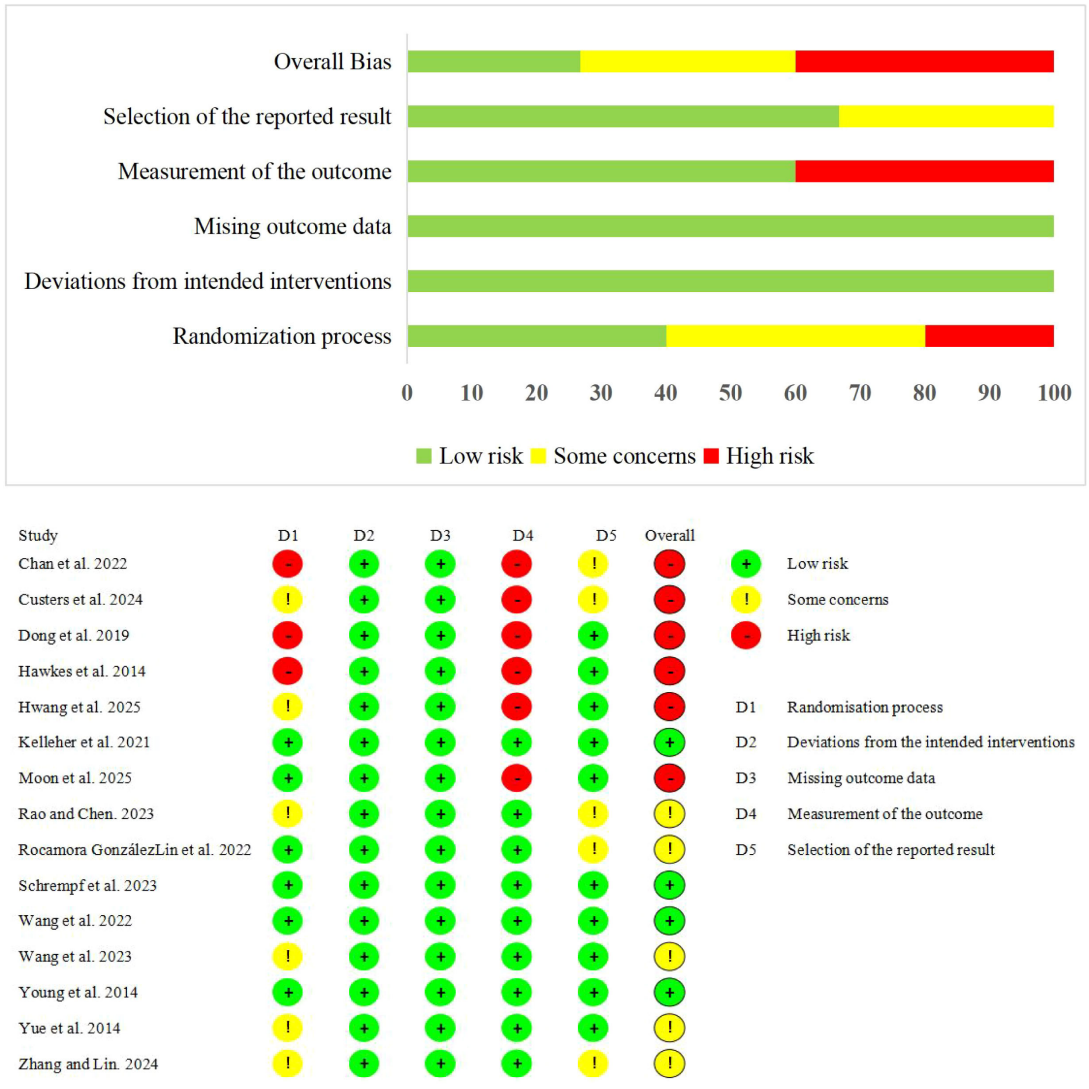
Summary of risk of bias.

The methodological quality of the included studies is detailed in **Figure 2**. Of the 15 studies, 6 were assessed as having a high risk of bias, 5 raised some concerns, and 4 were deemed to have a low risk of bias. Across the specific domains, the randomization process had the most varied results, with 6 studies at low risk, 6 with some concerns, and 3 at high risk. In contrast, both deviations from intended interventions and missing outcome data were consistently at low risk in all studies. The measurement of the outcome was rated as low risk in 9 studies and high risk in 6. Finally, regarding the selection of the reported result, 10 studies were at low risk, while 5 raised some concerns.

### 3.3 Meta-analysis results

#### 3.3.1 Effect of digital health interventions on anxiety

**Figure 3:**
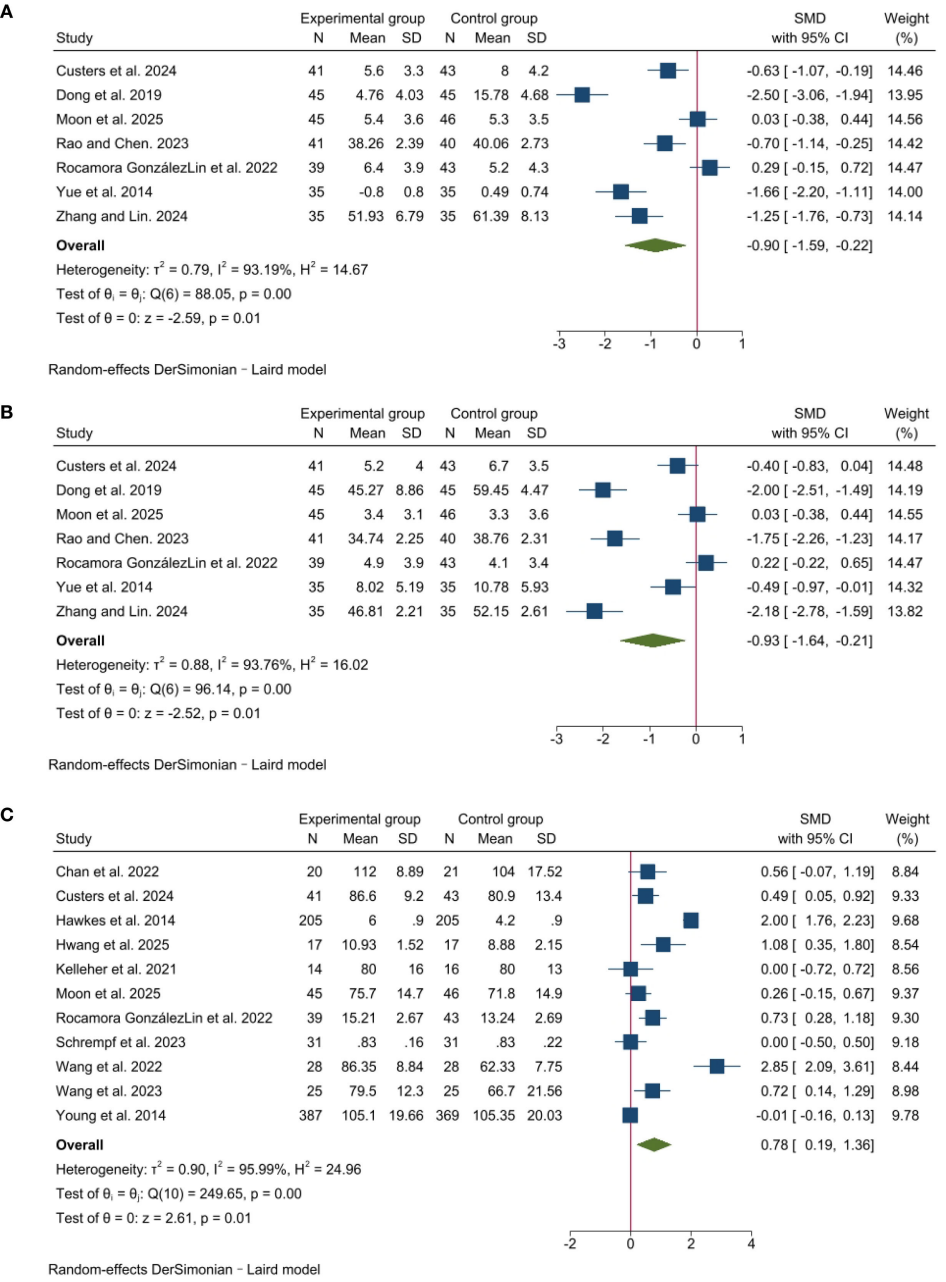
The effects of digital health interventions on anxiety, depression, and quality of life in CRC patients. **(A)** anxiety; **(B)** depression; **(C)** quality of life.

7 trials provided sufficient data on the effect of digital health interventions on anxiety among CRC patients. As shown in **Figure 3A**, the pooled results indicated a statistically significant reduction in anxiety of CRC patients compared to the control group (SMD = -0.90, 95% CI: -1.59 to -0.22; I² = 93%), with a 95% PI ranging from -3.36 to 1.56. The forest plot showed that the intervention effect favored the digital health group in most studies, although the confidence intervals were wide and heterogeneity was substantial.

#### 3.3.2 Effect of digital health interventions on depression

7 studies with a total of 568 participants compared depression levels between digital health interventions and control groups. The meta-analysis (**Figure 3B**) revealed a significant effect of digital health interventions on depression among CRC patients (SMD = -0.93, 95% CI: -1.64 to -0.21; I² = 94%), with a 95% PI ranging from -3.51 to 1.66. The forest plot illustrated a consistent direction of benefit for digital health interventions on depression across the majority of included studies, though the overall estimate is imprecise and heterogeneity is high.

#### 3.3.3 Effect of digital health interventions on quality of life

11 studies involving 1690 participants were employed to compare quality of life between the digital health interventions and control groups. The pooled results demonstrated that CRC patients receiving digital health interventions reported significantly higher quality of life than those in control groups (SMD = 0.78, 95% CI: 0.19 to 1.36, I² = 96%; **Figure 3C**), with a 95% PI ranging from -1.48 to 3.03. The forest plot showed that most study favored the intervention group, and the pooled effect size was positive, but the high heterogeneity suggests considerable variability across studies.

### 3.4 Subgroup analysis results

#### 3.4.1 Subgroup analysis for anxiety

Subgroup analyses for anxiety revealed that human-supported digital tools were associated with a large and significant reduction in anxiety (SMD = -1.33, 95% CI: -1.99 to -0.67), whereas self-guided tools showed no benefit (SMD = 0.15, 95% CI: -0.15 to 0.45), with a significant subgroup difference (P< 0.001). No significant subgroup differences were observed for therapeutic stage, intervention duration, outcome measurement tool, or risk of bias (all P > 0.05). Detailed subgroup results, including effect sizes, confidence intervals, and tests for subgroup differences, are presented in **Table 2** and **Supplementary Figure 1**.

**Table 2:** Subgroup analyses for anxiety.

Subgroups	Number of studies	SMD (95% CI)	Heterogeneity		P values across subgroups
			I²	P values	
Therapeutic stage					0.52
Active treatment	5	-1.01 (-2.02, 0.01)	95.44%	P< 0.001	
Survivors	2	-0.66 (-0.97, -0.35)	0%	0.83	
Intervention duration					0.47
≤ 6 weeks	3	-1.28 (-2.99, 0.43)	97.02%	P< 0.001	
> 6 weeks	4	-0.62 (-1.13, -0.11)	80.24%	P< 0.001	
Intervention format					P< 0.001
Self−guided digital tool	2	0.15 (-0.15, 0.45)	0%	0.39	
Human−supported digital tool	5	-1.33 (-1.99, -0.67)	88.58%	P< 0.001	
Outcome measurement tool					0.13
HADS-A	4	-0.48 (-1.25, 0.30)	91.41%	P< 0.001	
SAS	3	-1.47 (-2.50, -0.45)	91.91%	P< 0.001	
Risk of bias					0.06
High risk	3	-1.02 (-2.39, 0.34)	96.15%	P< 0.001	
Some concerns	2	-0.20 (-1.17, 0.76)	89.48%	P< 0.001	
Low risk	2	-1.44 (-1.84, -1.04)	11.13%	0.28	

#### 3.4.2 Subgroup analysis for depression

For depression, the subgroup analyses (**Table 3**; **Supplementary Figure 2**) identified intervention format and outcome measurement tool as significant moderators (P< 0.001). A large effect on depression was found for human-supported digital tools (SMD = -1.35, 95% CI: -2.11 to -0.59), in contrast to self-guided tools, which yielded no benefit (SMD = 0.12, 95% CI: -0.18 to 0.41). Moreover, the HADS-D showed no significant effect on depression (SMD = -0.15, 95% CI: -0.48 to 0.18), whereas the SDS demonstrated a very large effect (SMD = -1.96, 95% CI: -2.27 to -1.65). No other subgroup differences reached statistical significance (therapeutic stage, intervention duration, or risk of bias, all P > 0.05).

**Table 3:** Subgroup analyses for depression.

Subgroups	Number of studies	SMD (95% CI)	Heterogeneity		P values across subgroups
			I²	P values	
Therapeutic stage					0.82
Active treatment	5	-0.87 (-1.82, 0.07)	94.90%	P< 0.001	
Survivors	2	-1.06 (-2.39, 0.26)	93.54%	P< 0.001	
Intervention duration					0.71
≤ 6 weeks	3	-0.75 (-2.02, 0.51)	95.32%	P< 0.001	
> 6 weeks	4	-1.06 (-2.06, -0.05)	94.28%	P< 0.001	
Intervention format					P< 0.001
Self−guided digital tool	2	0.12 (-0.18, 0.41)	0%	0.54	
Human−supported digital tool	5	-1.35 (-2.11, -0.59)	91.31%	P< 0.001	
Outcome measurement tool					P< 0.001
HADS-D	4	-0.15 (-0.48, 0.18)	55.04%	0.08	
SDS	3	-1.96 (-2.27, -1.65)	0%	0.54	
Risk of bias					0.85
High risk	3	-0.78 (-1.93, 0.36)	94.85%	P< 0.001	
Some concerns	2	-0.76 (-2.68, 1.17)	96.93%	P< 0.001	
Low risk	2	-1.33 (-2.99, 0.33)	94.70%	P< 0.001	

#### 3.4.3 Subgroup analysis for quality of life

As shown in **Table 4** and **Supplementary Figure 3**, all subgroup factors (therapeutic stage, intervention duration, intervention format, outcome measurement tool, and risk of bias) significantly modified the effects of digital health interventions on quality of life (all P< 0.001). Among survivors (SMD = 1.10, 95% CI: 0.29 to 1.92), digital health interventions showed a large effect size on quality of life, whereas the effect was not significant among patients undergoing active treatment (SMD = 0.34, 95% CI: -0.03 to 0.71). Moreover, a significant effect was observed for longer interventions (> 6 weeks) (SMD = 0.96, 95% CI: 0.14 to 1.78, but not for shorter ones (≤ 6 weeks) (SMD = 0.44, 95% CI: -0.06 to 0.94). Subgroup analysis by intervention format demonstrated that the effect size of human-supported digital tools (SMD = 0.82, 95% CI: 0.04 to 1.59) was significantly larger than that of self-guided tools (SMD = 0.62, 95% CI: 0.18 to 1.06). The EORTC-QLQ-C30 showed a significantly large effect (SMD = 1.06, 95% CI: 0.26 to 1.86), whereas the FACT-C (SMD = 0.83, 95% CI: -0.59 to 2.24) and other scales (SMD = 0.28, 95% CI: -0.24 to 0.80) did not reach significance. Regarding risk of bias, high-risk (SMD = 0.88, 95% CI: 0.04 to 1.73) and some concerns studies (SMD = 0.72, 95% CI: 0.37 to 1.08) showed a significant effect, but low-risk studies did not (SMD = 0.67, 95% CI: -0.32 to 1.65).

**Table 4:** Subgroup analyses for quality of life.

Subgroups	Number of studies	SMD (95% CI)	Heterogeneity		P values across subgroups
			I²	P values	
Therapeutic stage					P< 0.001
Active treatment	5	0.34 (-0.03, 0.71)	77.30%	P< 0.001	
Survivors	6	1.10 (0.29, 1.92)	93.90%	P< 0.001	
Intervention duration					P< 0.001
≤ 6 weeks	4	0.44 (-0.06, 0.94)	66.59%	0.03	
> 6 weeks	7	0.96 (0.14, 1.78)	97.50%	P< 0.001	
Intervention format					P< 0.001
Self−guided digital tool	3	0.62 (0.18, 1.06)	55.44%	0.11	
Human−supported digital tool	8	0.82 (0.04, 1.59)	97.14%	P< 0.001	
Outcome measurement tool					P< 0.001
EORTC-QLQ-C30	5	1.06 (0.26, 1.86)	88.84%	P< 0.001	
FACT-C	3	0.83 (-0.5, 2.24)	99.01%	P< 0.001	
Other scales	3	0.28 (-0.24, 0.80)	63.84%	0.06	
Risk of bias					P< 0.001
High risk	5	0.88 (0.04, 1.73)	94.77%	P< 0.001	
Some concerns	2	0.72 (0.37, 1.08)	0%	0.98	
Low risk	4	0.67 (-0.32, 1.65)	94.35%	P< 0.001	

### 3.5 Sensitivity analyses and publication bias

Leave-one-out sensitivity analyses demonstrated the robustness of the pooled results for anxiety (**Supplementary Table 2**), depression (**Supplementary Table 3**), and quality of life (**Supplementary Table 4**). Specifically, excluding any single study did not meaningfully alter the pooled effect sizes: the effect sizes for anxiety (SMD: -1.10 to -0.64), depression (SMD: -1.12 to -0.72), and quality of life (SMD: 0.62 to 0.86) all remained statistically significant. The I² values calculated across all iterations did not decrease substantially, indicating that the high heterogeneity was not attributable to any individual study. No significant publication bias was detected for the quality of life outcome, as assessed by the funnel plot (**Supplementary Figure 4**) and Egger’s test (**Supplementary Figure 5**).

### 3.6 The certainty of evidence

According to the GRADE framework, the certainty of evidence was low for digital health interventions on reducing anxiety and depression and improving quality of life in CRC patients. The downgrading was primarily due to a serious risk of bias (most included studies were rated as having moderate or high risk of bias) and a serious inconsistency (substantial heterogeneity was observed across studies, with I² > 50% for all three outcomes). Therefore, despite the statistical significance, our confidence in the effect estimates remains limited. A detailed summary of these ratings and the underlying rationale is presented in **Supplementary Table 5**.

## 4 Discussion

This meta-analysis of 15 randomized controlled trials, comprising 2018 participants, examined the effects of digital health interventions on anxiety, depression, and quality of life in CRC patients. The pooled findings indicated that digital health interventions have a potential beneficial effect on alleviating anxiety and depression while enhancing quality of life in this population, compared with the control groups. However, the relatively large effect sizes observed should be interpreted with caution, as they may be subject to overestimation due to small-study effects (several included trials had limited sample sizes) and methodological biases (such as lack of blinding, inadequate allocation concealment in high risk of bias studies). Moreover, the extremely high heterogeneity represents a critical issue that substantially weakens the reliability of the pooled estimates. This inconsistency likely arises from multiple factors, including differences in therapeutic stage, intervention duration, intervention formats, outcome measurement tools, and risk of bias. Subgroup analyses could not fully account for the observed variability, partly due to the limited number of studies within each subgroup. Consequently, the findings should be considered preliminary and tentative, and the overall certainty of evidence is low. The substantial inconsistency also limits the generalizability of these findings to the broader CRC population. Therefore, further high-quality randomized controlled trials are warranted to establish more robust conclusions.

Our findings indicated that digital health interventions may be associated with reduced anxiety and depression in CRC patients. However, given the high heterogeneity and low certainty of evidence, these results should not be overinterpreted as definitive proof of effectiveness. The alleviation of anxiety and depression in CRC patients following digital health interventions may be attributed to multiple psychological pathways. First, digital health interventions reduce psychological distress by providing vicarious experiences via peer supports on social media platforms, and motivational feedback from healthcare professionals (39). Moreover, the electronic symptom management tools embedded in digital platforms assist patients in reevaluating the seriousness of their cancer threat and offer them practical coping strategies (40). Finally, digital health interventions address patients’ need for autonomy, build competence through visualized progress, and foster relatedness via online communities (41). The fulfillment of these psychological needs is a crucial prerequisite for improving mental health. Although these findings are encouraging, further research is still needed to explore how digital health interventions can be optimized based on individual patient characteristics, with the aim of achieving more personalized psychological support. Notably, subgroup analyses revealed that human-supported digital health interventions were associated with potential reduction in anxiety and depression among CRC patients, whereas self-guided digital tools showed no significant effect. This discrepancy may be explained by the fact that human-supported interventions provide personalized feedback, emotional encouragement, and accountability through regular coach or clinician interaction, which are critical for addressing the complex psychological needs of cancer patients (39). In contrast, self-guided tools, although more scalable, often lack real-time adaptation and empathetic support, potentially limiting their efficacy for mood-related outcomes. Furthermore, when the SDS was used as the outcome measurement tool, digital health interventions demonstrated a significant effect on reducing depression in CRC patients, whereas no significant effect was observed when the HADS-D was used. This discrepancy may be attributable to the differing item composition of the two scales. The SDS places greater emphasis on somatic symptoms, such as sleep disturbance, fatigue, and appetite loss, which are more directly modifiable by digital health interventions like symptom monitoring and physical activity promotion (42). In contrast, the HADS-D was specifically designed to exclude somatic items to avoid confounding with physical illness, focusing instead on anhedonia and cognitive-affective symptoms (43). These latter symptoms may be less responsive to the types of digital interventions included in this meta-analysis.

Our findings demonstrated that digital health interventions may be associated with improved quality of life in CRC patients. According to previously established conversion algorithms, an SMD of 0.78 corresponds to an approximate mean difference of 8–10 points on the EORTC-QLQ-C30 (28). As the minimal important difference (MID) for CRC patients has been reported to range from 5 to 10 points (44), the observed effect exceeds the lower threshold of the MID, suggesting that the improvement may be clinically meaningful. However, the interpretation of this effect is complicated by substantial heterogeneity and low certainty of evidence. All five subgroup factors significantly modified the effect on quality of life. Survivors, longer intervention duration, human-supported tools, the EORTC-QLQ-C30, and high-risk studies each showed larger or significant effects compared with their counterparts. Despite these significant subgroup differences, substantial residual heterogeneity persisted within most subgroups, indicating that unmeasured factors such as patient age, comorbidity, adherence, cultural context, and intervention intensity also contribute to inconsistency. Taken together, these findings indicate that the effect on quality of life is highly context-dependent and should be interpreted with caution. Notably, the fact that only high-risk studies showed a significant effect raises the possibility that methodological biases may have inflated the observed effect sizes. Therefore, the present evidence is hypothesis-generating rather than definitive. The potential mechanisms underlying the improvement in quality of life include real-time symptom monitoring, medication adherence support, and social connection. Digital health tools empower patients to monitor common symptoms such as pain, fatigue, and nausea in real time (40), providing immediate feedback and triggering alerts to healthcare professionals when abnormal data is detected. This enables timely pharmacological or nursing interventions before symptoms worsen, thereby alleviating physical suffering (45). In addition, digital health interventions support patients in adhering to scheduled treatments and follow-up visits by providing medication reminders and appointment notifications, which optimizes therapeutic outcomes and indirectly improves long-term quality of life (46). Finally, participating in online communities or patient groups connects CRC patients with others who have shared experiences, providing a platform for emotional expression and mutual learning (47). This social support serves as a buffer against the psychosocial distress associated with cancer, positively influencing the psychological and social aspects of quality of life. Therefore, integrating digital health interventions into CRC management may provide a cost-effective solution to enhance quality of life. However, the potential clinical significance noted above, the substantial heterogeneity and low certainty of evidence warrant cautious interpretation, and future studies should use validated anchor-based methods to confirm the minimal clinically important difference for digital health interventions in this population.

Beyond statistical findings, several practical factors may influence the effectiveness of digital health interventions in real-world. First, low adherence could reduce benefits, while usability issues such as complex interfaces or technical glitches may hinder engagement, especially among older or less tech-savvy patients (48). Second, digital literacy is a prerequisite, as lower education or limited smartphone access may widen disparities, and accessibility barriers like device costs, internet connectivity, and lack of technical support affect equitable implementation (49). Third, cross-cultural differences also matter because attitudes toward technology and mental health stigma vary, yet most studies were conducted in high-income countries, leaving applicability elsewhere unclear (50). Finally, cost-effectiveness has not been formally evaluated, but potential resource savings exist, so formal economic analyses are needed.

## 5 Limitations

Several limitations should be considered. First, the restriction of included studies to those published in English and Chinese may have introduced language bias, and the absence of grey literature searching may have further increased the risk of publication bias. Both limitations constrain the external validity of our findings. Second, for anxiety and depression, the limited number of included studies precludes a robust assessment of publication bias. For quality of life, although the number of studies marginally met the recommended threshold, the power of Egger’s test remains limited. Therefore, for all outcomes, the absence of evidence for publication bias should be interpreted with caution, and the possibility of residual bias can’t be excluded. Furthermore, significant heterogeneity was not fully explained by subgroup analyses, likely owing to the small number of studies per subgroup and inconsistent reporting of moderators, while the absence of meta-regression adds another limitation in explaining the observed inconsistency. Additionally, based on the GRADE approach, the certainty of evidence was rated as low, suggesting that our findings should be considered hypothesis-generating rather than definitive. Finally, as follow-up data were generally lacking in the included studies, the evaluation of long-term benefits was not feasible.

## 6 Conclusion

In conclusion, the findings of this meta-analysis suggested that digital health interventions may offer potential benefits in reducing anxiety and depression and improving quality of life in CRC patients, with moderate to large effect sizes. However, these effects should be interpreted with caution due to substantial heterogeneity across studies and low certainty of evidence. From a clinical perspective, digital health interventions could be considered as a complementary approach for CRC patients, particularly for those with limited access to in-person support. Future high-quality, well-powered randomized controlled trials with standardized outcome measures and longer follow-up are urgently needed to confirm these potential benefits, identify which patient subgroups are most likely to respond, and inform the development of evidence-based guidelines.
