# Supplementary material for: Effects of digital health interventions on anxiety, depression, and quality of life in colorectal cancer patients: a meta-analysis of randomized controlled trials
Source: Front Oncol. 2026 Jun 2;16:1832924. doi: 10.3389/fonc.2026.1832924 (PMC13268989; doi:10.3389/fonc.2026.1832924)
Supplement: Supplementary file 1 [file DataSheet1.docx]

**Supplemental Materials**

Table S1. The search strategies.

| Databases | Step | Search Strategies |
| --- | --- | --- |
| PubMed | #1 | "colorectal [neoplasms](https://www.ncbi.nlm.nih.gov/mesh/68009369)"[Mesh Terms] Sort by: Best match |
|  | #2 | "colorectal cancer"[Title/Abstract] OR "colon cancer"[Title/Abstract] OR "rectal cancer"[Title/Abstract] Sort by: Best match |
|  | #3 | "digital health"[Mesh Terms] Sort by: Best match |
|  | #4 | "telemedicine"[Mesh Terms] Sort by: Best match |
|  | #5 | "eHealth"[Title/Abstract] OR "mHealth"[Title/Abstract] OR "website"[Title/Abstract] OR "app"[Title/Abstract] OR "phone"[Title/Abstract] OR "email"[Title/Abstract] OR "text message"[Title/Abstract] OR "social media"[Title/Abstract] OR "virtual reality"[Title/Abstract] OR "wearable devices"[Title/Abstract] Sort by: Best match |
|  | #6 | "RCT"[Title/Abstract] OR "randomized clinical trial"[Title/Abstract] OR "randomized controlled trial"[Title/Abstract] OR "randomized trial"[Title/Abstract] OR "randomised controlled trial"[Title/Abstract] OR "randomised trial"[Title/Abstract] Sort by: Most Recent |
|  | #7 | #1 OR #2 Sort by: Best match |
|  | #8 | #3 OR #4 OR #5 Sort by: Best match |
|  | #9 | #6 AND #7 AND #8 Sort by: Best match |
| Web of Science | #1 | **TS=(**colorectal [neoplasms](https://www.ncbi.nlm.nih.gov/mesh/68009369)**) OR TS=(**colorectal **cancer**) OR TS=(colon cancer) OR TS=(rectal cancer) |
|  | #2 | **Ts=(**digital health**) OR Ts=(**telemedicine**) OR** TS=(eHealth) **OR** TS=(mHealth) **OR** TS=(website) **OR Ts=(app) OR** TS=(phone) **OR** TS=(email) **OR** TS=(text message) **OR** TS=(social media) **OR** TS=(virtual reality) **OR** TS=(wearable devices) |
|  | #3 | TS=(RCT) OR **TS=(**randomized clinical trial**) OR TS=(**randomized controlled trial) OR TS=(randomized trial) OR TS=(randomised controlled trial) OR TS=(randomised trial) |
|  | #4 | #1 AND #2 AND #3 |
| Scopus | #1 | ABS("colorectal [neoplasms](https://www.ncbi.nlm.nih.gov/mesh/68009369)" OR "colorectal cancer" OR "colon cancer" OR "rectal cancer") |
|  | #2 | ABS("digital health" OR "telemedicine" OR "eHealth" OR "mHealth" OR "website" OR "app" OR "phone" OR "email" OR "text message" OR "social media" OR "virtual reality" OR "wearable devices") |
|  | #3 | ABS ("RCT" OR "randomized clinical trial" OR "randomized controlled trial" OR "randomized trial" OR "randomised controlled trial OR "randomised trial) |
|  | #4 | #1 AND #2 AND #3 |
| Embase | **#1** | **'**colorectal cancer**'/exp** |
|  | **#2** | 'colorectal cancer':ti,ab,kw OR 'colon cancer':ti,ab,kw OR 'rectal cancer':ti,ab,kw |
|  | **#3** | **'digital health'/exp** |
|  | **#4** | 'digital health**':ti,ab,kw OR 'telemedicine':ti,ab,kw OR 'eHealth':ti,ab,kw OR 'mHealth':ti,ab,kw OR 'website':ti,ab,kw OR 'app':ti,ab,kw OR 'phone':ti,ab,kw OR 'email':ti,ab,kw OR 'text message':ti,ab,kw OR 'social media':ti,ab,kw OR '**virtual reality**':ti,ab,kw OR '**wearable devices**':ti,ab,kw** |
|  | **#5** | 'RCT**':ti,ab,kw OR '**randomized clinical trial**':ti,ab,kw OR '**randomized controlled trial**':ti,ab,kw OR '**randomized trial**':ti,ab,kw OR '**randomised controlled trial**':ti,ab,kw OR '**randomised trial**':ti,ab,kw** |
|  | #6 | #1 OR #2 |
|  | #7 | #3 OR #4 |
|  | #8 | #5 AND #6 AND #7 |
| Cochrane Library | #1 | MeSH descriptor: [Colorectal Neoplasms] explode all trees |
|  | #2 | (colorectal cancer):ti,ab,kw OR (colon cancer):ti,ab,kw OR (rectal cancer):ti,ab,kw |
|  | #3 | MeSH descriptor: [Digital Health] explode all trees |
|  | #4 | 'digital health**':ti,ab,kw OR 'telemedicine':ti,ab,kw OR 'eHealth':ti,ab,kw OR 'mHealth':ti,ab,kw OR 'website':ti,ab,kw OR 'app':ti,ab,kw OR 'phone':ti,ab,kw OR 'email':ti,ab,kw OR 'text message':ti,ab,kw OR 'social media':ti,ab,kw OR '**virtual reality**':ti,ab,kw OR '**wearable devices**':ti,ab,kw** |
|  | #5 | (RCT)**:ti,ab,kw OR (**randomized clinical trial)**:ti,ab,kw OR (**randomized controlled trial)**:ti,ab,kw OR (**randomized trial)**:ti,ab,kw OR (**randomised controlled trial)**:ti,ab,kw OR (**randomised trial)**:ti,ab,kw** |
|  | #6 | #1 OR #2 |
|  | #7 | #3 OR #4 |
|  | #8 | #5 AND #6 AND #7 |
| CINAHL | S1 | **TI** colorectal [neoplasms](https://www.ncbi.nlm.nih.gov/mesh/68009369) **OR TI** colorectal **cancer** OR TI colon cancer OR TI rectal cancer |
|  | S2 | **TI** digital health **OR TI** telemedicine **OR** TI eHealth **OR TI** mHealth **OR** TI website **OR TI app OR** TI phone **OR** TI email **OR** TI text message **OR** TI social media **OR** TI virtual reality **OR** TI wearable devices |
|  | S3 | TI RCT **OR TI** randomized clinical trial **OR TI** randomized controlled trial **OR TI** randomized trial **OR TI** randomised controlled trial **OR TI** randomised trial |
|  | S4 | #1 AND #2 AND #3 |
| CNKI |  | (SU="结直肠癌" + "结肠癌" + "直肠癌") AND (SU="电话" + "短信" + "互联网" + "app" + "视频" + "网站" + "信息平台" + "远程护理" + "远程康复") |
| WanFang |  | 题名或关键词:(结直肠癌 OR 结肠癌 OR 直肠癌) and 题名或关键词:(电话 OR 短信 OR 互联网 OR APP OR 视频 OR 网站 OR 信息平台 OR远程护理 OR 远程康复) |
| VIP |  | (题名或关键词= 结直肠癌 OR 结肠癌 OR 直肠癌) and (题名或关键词=电话 OR 短信 OR 互联网 OR APP OR 视频 OR 网站 OR 信息平台 OR远程护理 OR 远程康复) |
| CBM |  | ("结直肠癌"[常用字段:智能] OR "结肠癌"[常用字段:智能] OR "直肠癌"[常用字段:智能] )AND ("电话"[常用字段:智能] OR "短信"[常用字段:智能] OR "互联网"[常用字段:智能] OR "APP"[常用字段:智能] OR "视频"[常用字段:智能] OR "网站"[常用字段:智能] OR "信息平台"[常用字段:智能] OR "远程医疗"[常用字段:智能] ) |


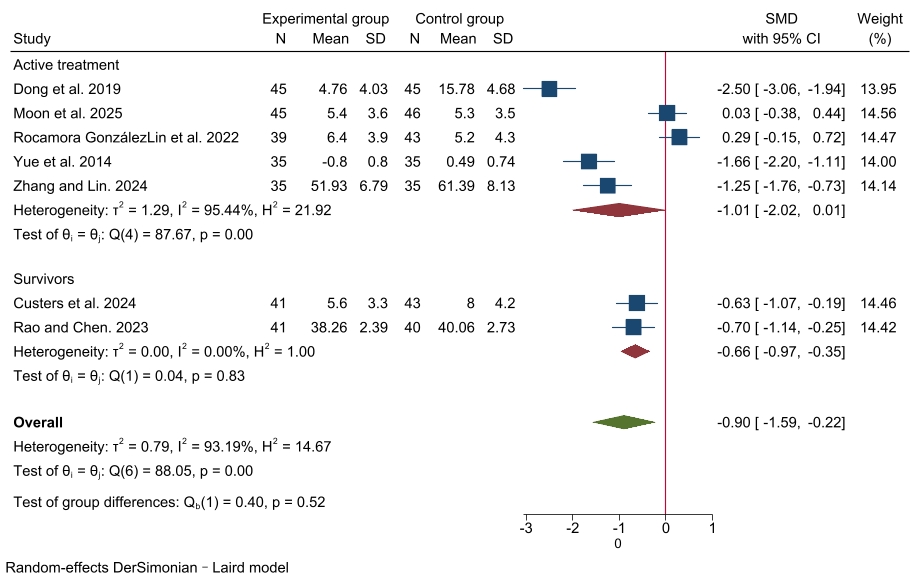
(a)


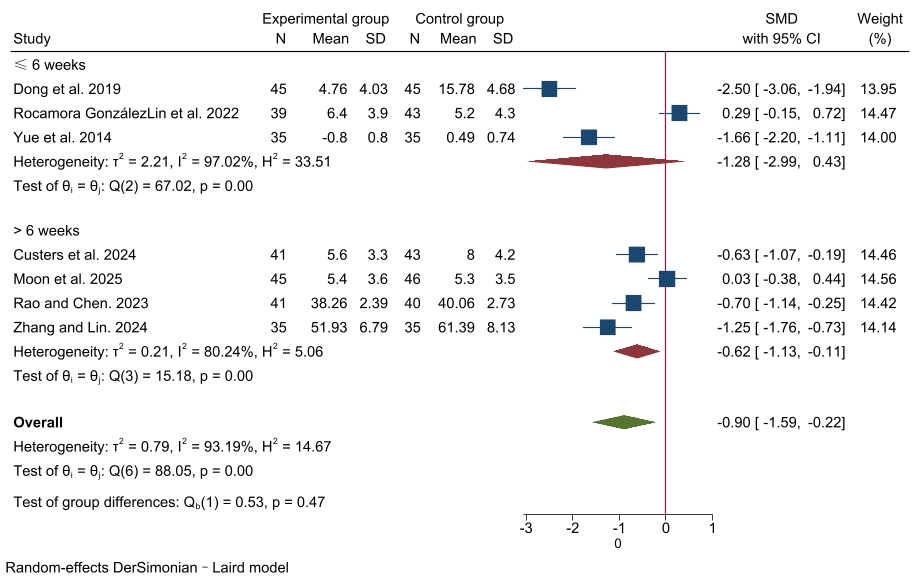


(b)


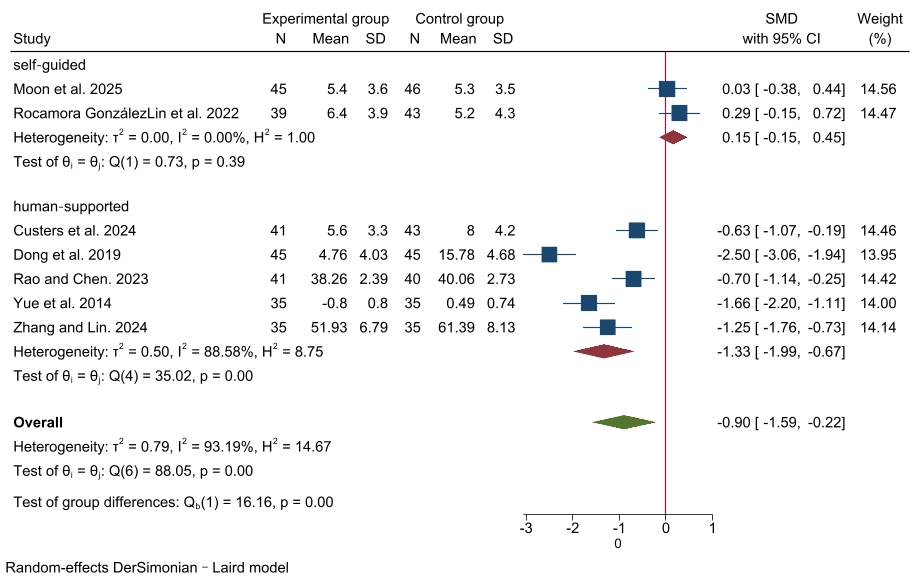


(c)


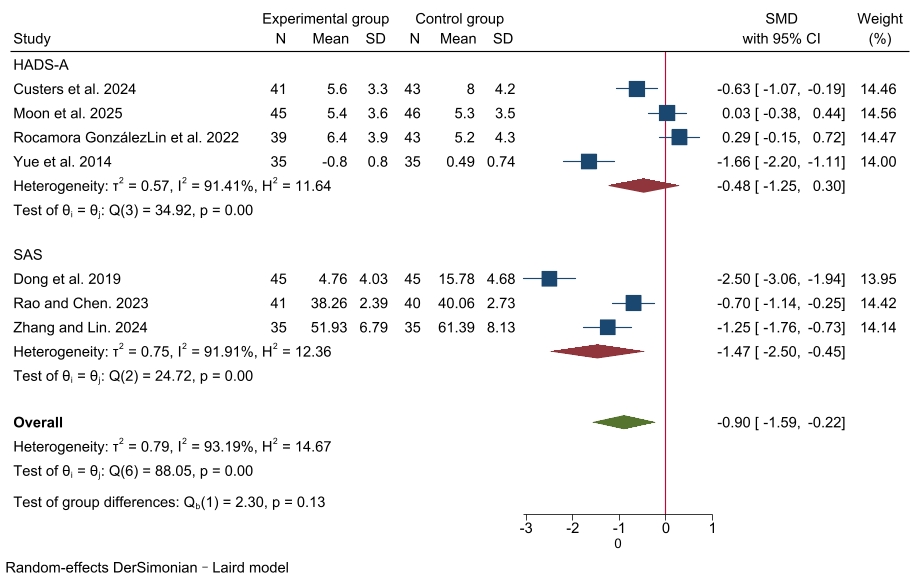


(d)


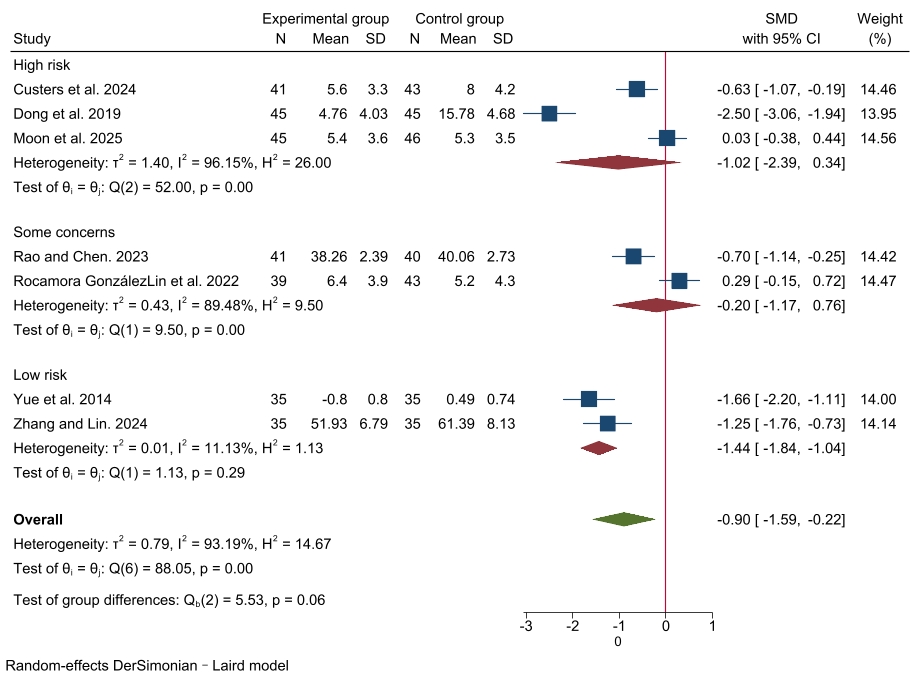
(e)

Figure S1. The subgroup analysis for anxiety. (a) Based on therapeutic stage; (b) Based on intervention duration; (c) Based on intervention format; (d) Based on outcome measurement tool; (e) Based on risk of bias.

**
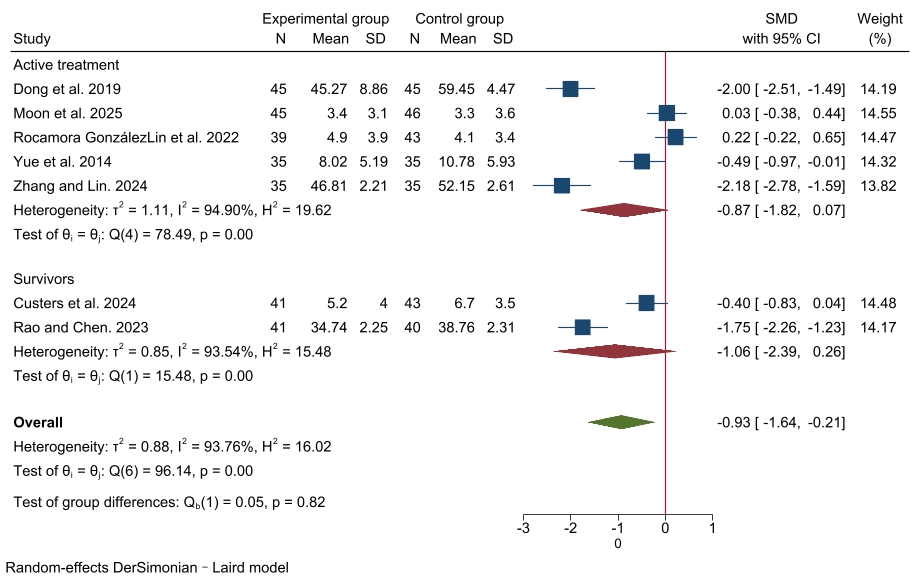
**

(a)


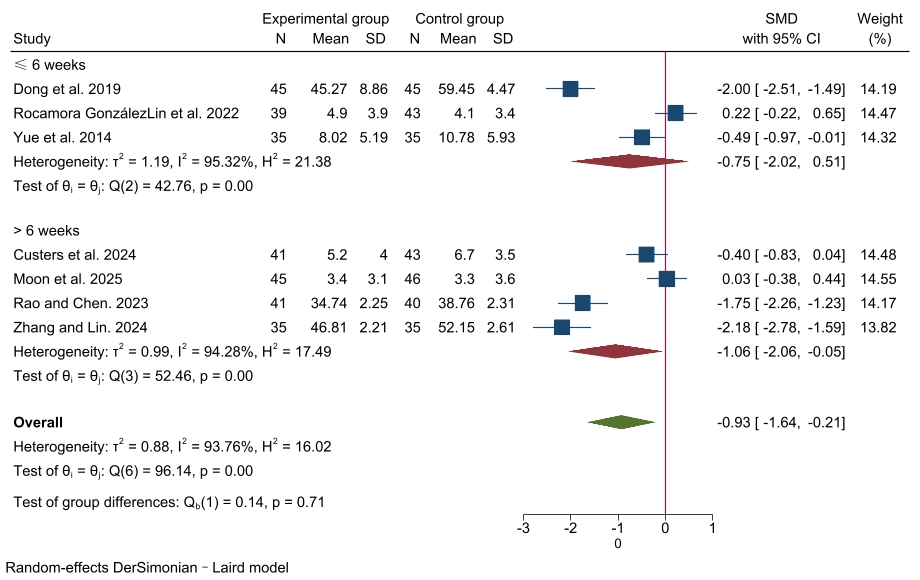


(b)


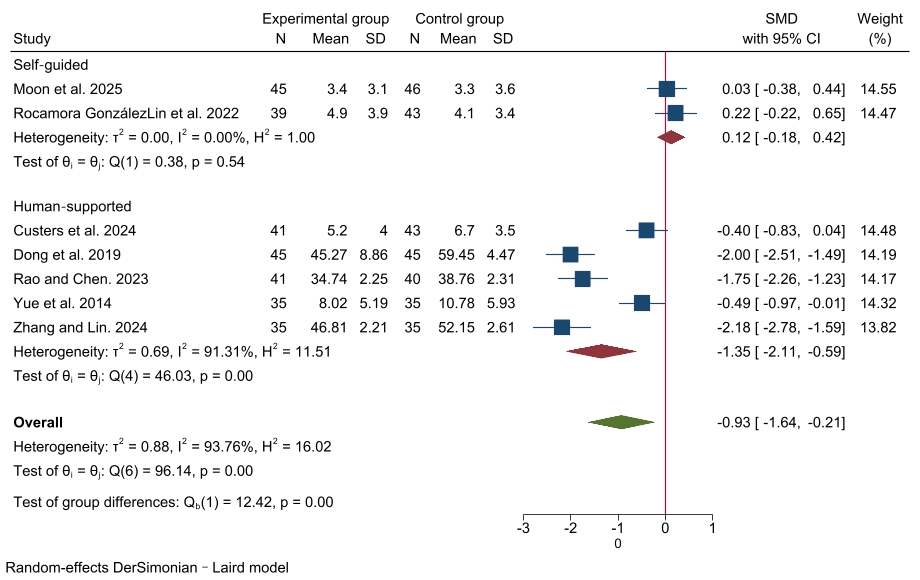


(c)


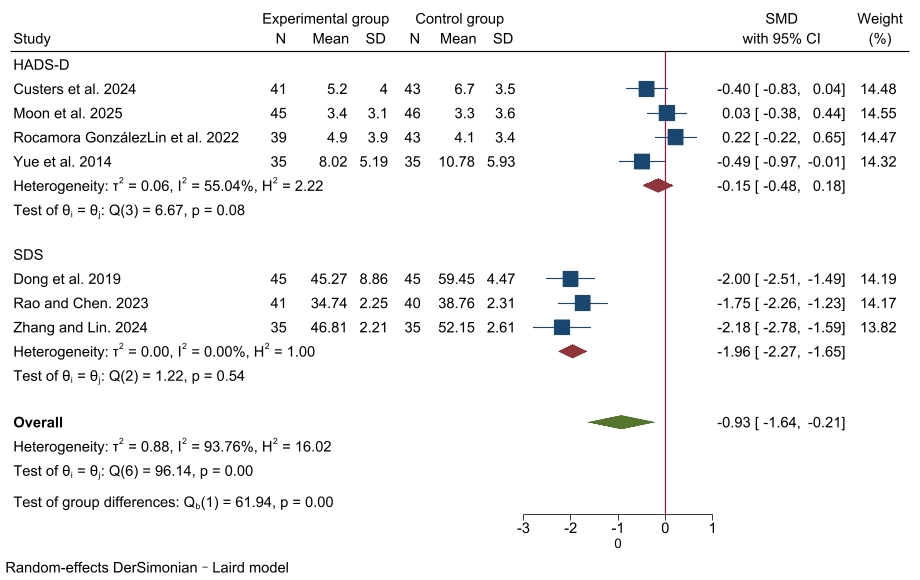


(d)


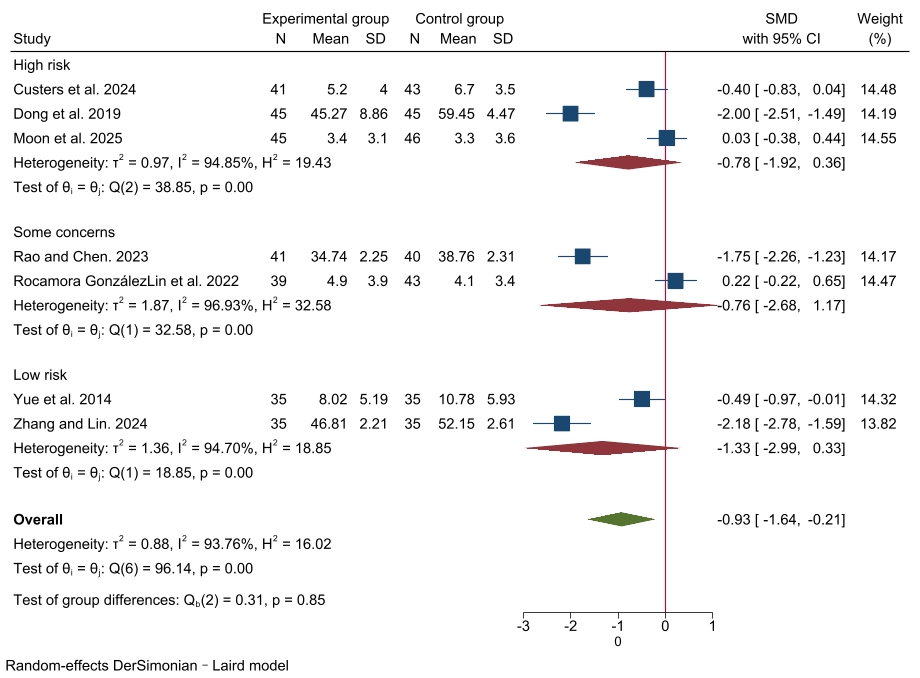


(e)

Figure S2. The subgroup analysis for depression. (a) Based on therapeutic stage; (b) Based on intervention duration; (c) Based on intervention format; (d) Based on outcome measurement tool; (e) Based on risk of bias.

**
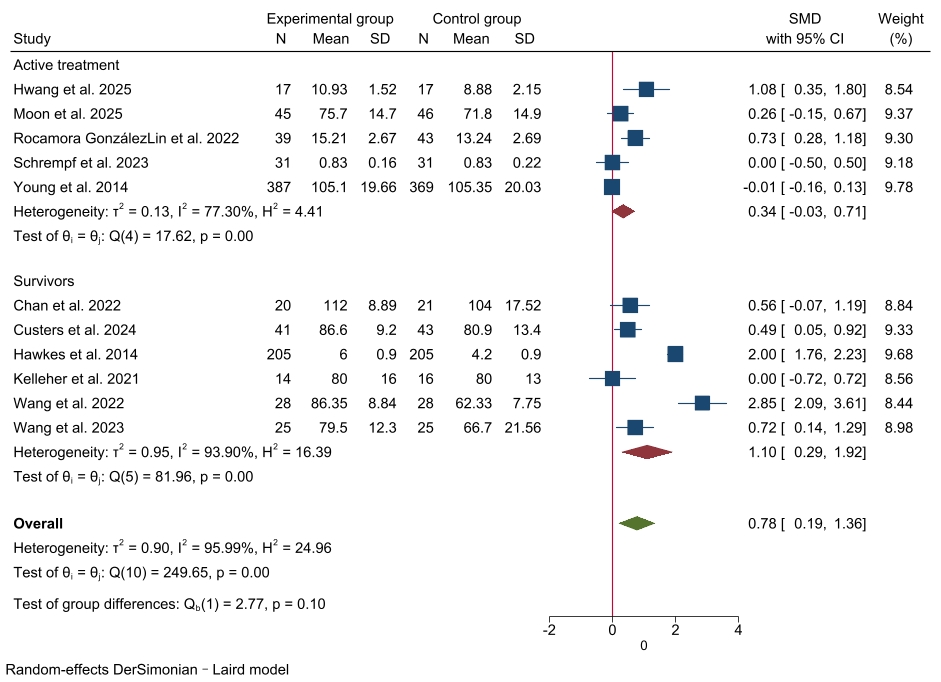
**

(a)


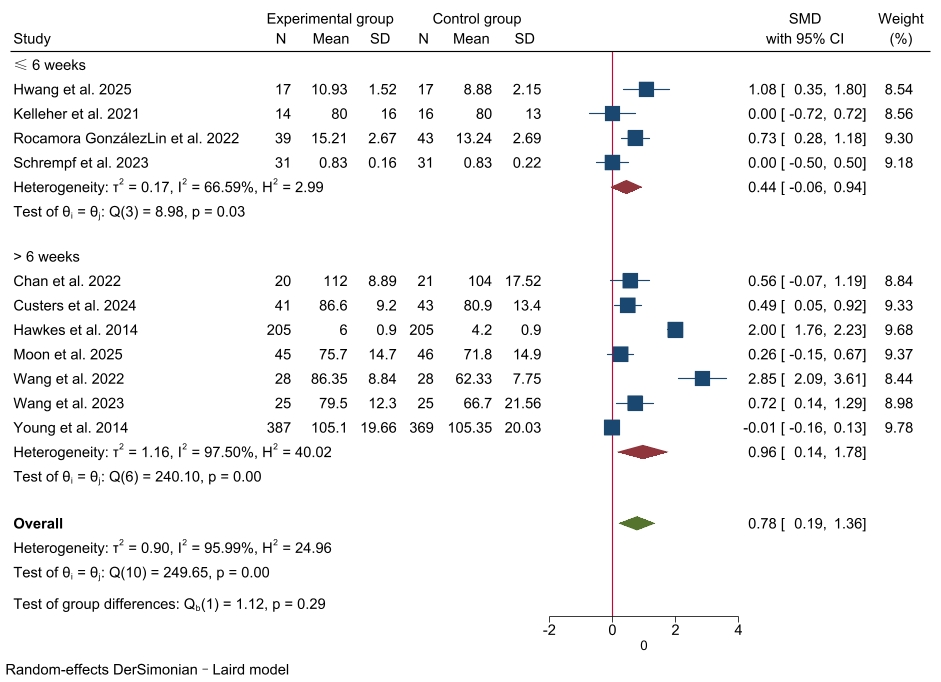


(b)


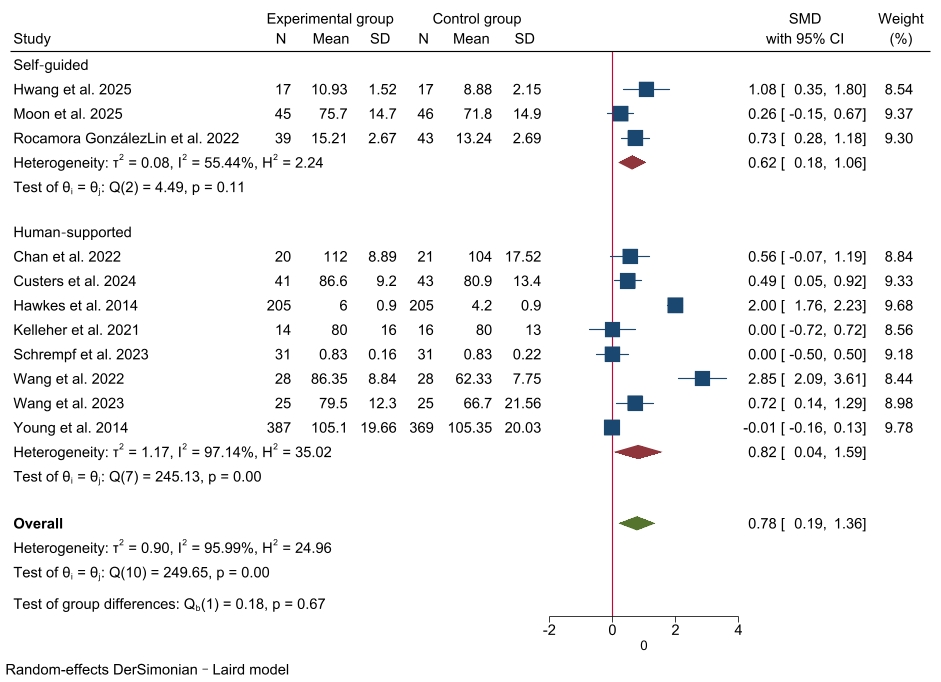


(c)


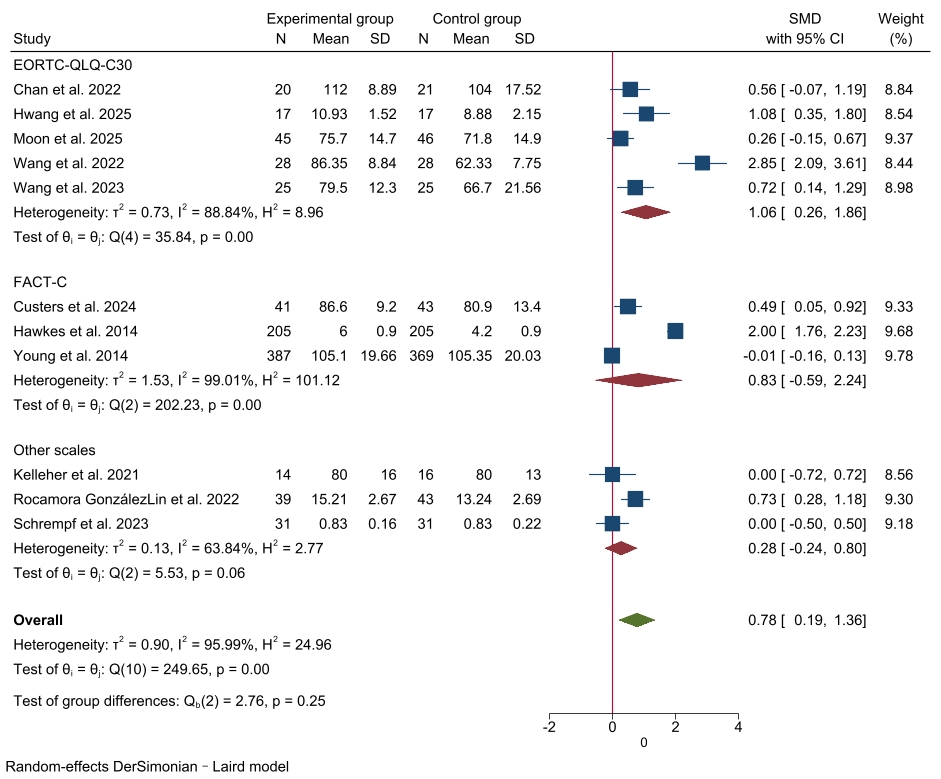


(d)


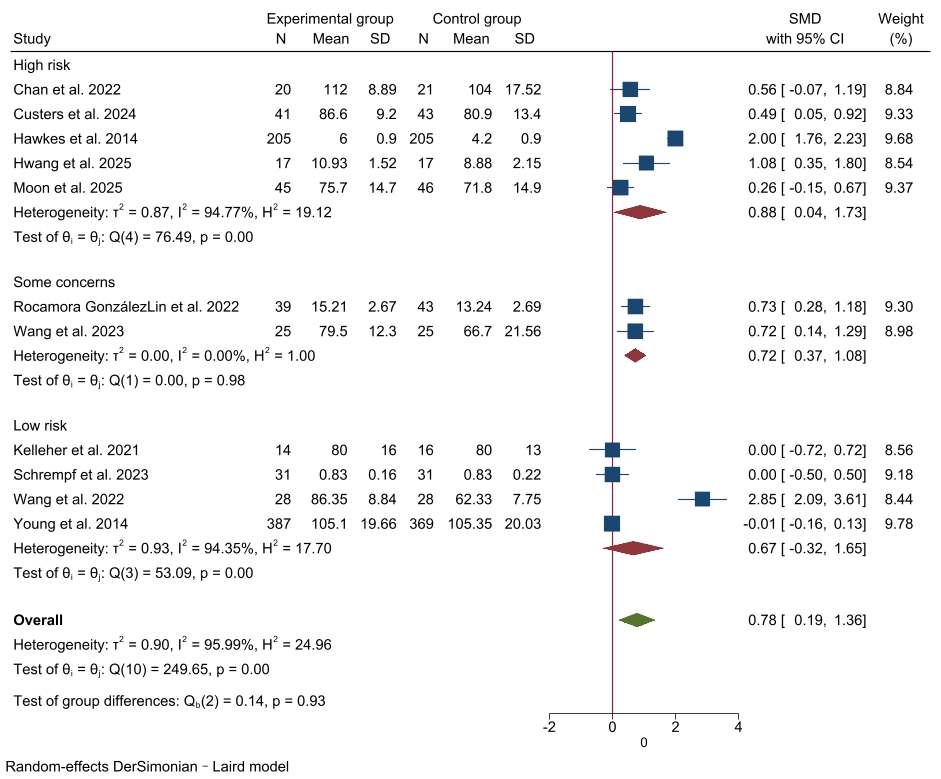


(e)

Figure S3. The subgroup analysis for quality of life. (a) Based on therapeutic stage; (b) Based on intervention duration; (c) Based on intervention format; (d) Based on outcome measurement tool; (e) Based on risk of bias.

Table S2. The results of the sensitivity analysis for anxiety.

| **Omitted Study** | **SMD** | **95% CI** | **I²** |
| --- | --- | --- | --- |
| Custers et al. 2024 | -0.95 | (-1.77, -0.13) | 93.19% |
| Dong et al. 2019 | -0.64 | (-1.20, -0.07) | 88.92% |
| Moon et al. 2025 | -1.06 | (-1.81, -0.31) | 93.03% |
| Rao and Chen. 2023 | -0.94 | (-1.76, -0.12) | 94.32% |
| Rocamora GonzálezLin et al. 2022 | -1.10 | (-1.80, -0.41) | 92.00% |
| Yue et al. 2014 | -0.78 | (-1.52, -0.04) | 93.41% |
| Zhang and Lin. 2024 | -0.85 | (-1.63, -0.07) | 94.03% |

Table S3. The results of the sensitivity analysis for depression.

| **Omitted Study** | **SMD** | **95% CI** | **I²** |
| --- | --- | --- | --- |
| Custers et al. 2024 | -1.02 | (-1.87, -0.17) | 94.63% |
| Dong et al. 2019 | -0.75 | (-1.46, -0.03) | 92.80% |
| Moon et al. 2025 | -1.09 | (-1.88, -0.29) | 93.70% |
| Rao and Chen. 2023 | -0.79 | (-1.56, -0.02) | 93.72% |
| Rocamora GonzálezLin et al. 2022 | -1.12 | (-1.88, -0.36) | 93.20% |
| Yue et al. 2014 | -1.00 | (-1.85, -0.16) | 94.73% |
| Zhang and Lin. 2024 | -0.72 | (-1.43, -0.01) | 93.03% |

Table S4. The results of the sensitivity analysis for quality of life.

| **Omitted Study** | **SMD** | **95% CI** | **I²** |
| --- | --- | --- | --- |
| Chan et al. 2022 | 0.80 | (0.17, 1.42) | 96.39% |
| Custers et al. 2024 | 0.81 | (0.17, 1.45) | 96.39% |
| Hawkes et al. 2014 | 0.62 | (0.22, 1.03) | 87.74% |
| Hwang et al. 2025 | 0.75 | (0.13, 1.37) | 96.36% |
| Kelleher et al. 2021 | 0.85 | (0.23, 1.47) | 96.36% |
| Moon et al. 2025 | 0.83 | (0.19, 1.47) | 96.37% |
| Rocamora GonzálezLin et al. 2022 | 0.78 | (0.14, 1.42) | 96.38% |
| Schrempf et al. 2023 | 0.86 | (0.23, 1.48) | 96.33% |
| Wang et al. 2022 | 0.59 | (0.01, 1.16) | 95.78% |
| Wang et al. 2023 | 0.78 | (0.16, 1.41) | 96.39% |
| Young et al. 2014 | 0.86 | (0.28, 1.45) | 93.27% |


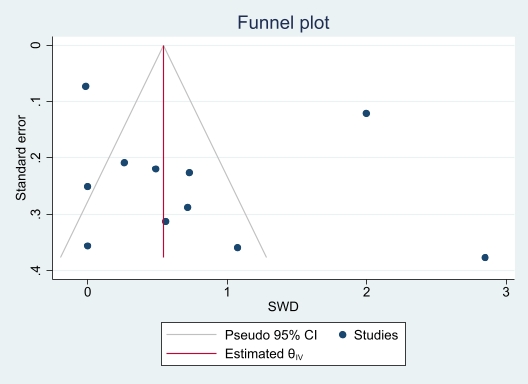


Figure S4. Funnel plot for quality of life.


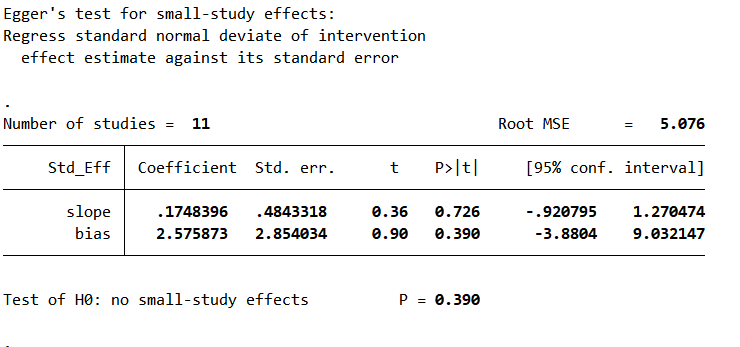
Figure S5. The result of Egger's test.

**Table S5.** GRADE summary of the quality of the evidence for the outcomes.

| Outcome | Quality assessment | | | | | No. of Participants  (studies) | Effect size (95% CI) | Quality of the evidence (GRADE) |
| --- | --- | --- | --- | --- | --- | --- | --- | --- |
|  | Risk of bias | Inconsistency | Indirectness | Imprecision | Other considerations |  |  |  |
| 1. Anxiety | Serious^a^ | Serious^b^ | No serious^c^ | No serious^d^ | None | 568 (7) | SMD = -0.90, [-1.59, -0.22] | ⨁⨁⭘⭘  Low |
| 1. Depression | Serious^a^ | Serious^b^ | No serious^c^ | No Serious^d^ | None | 568 (7) | SMD = -0.93, [-1.65, -0.21] | ⨁⨁⭘⭘  Low |
| 1. Quality of life | Serious^a^ | Serious^b^ | No serious^c^ | No serious^d^ | None | 756 (11) | SMD = 0.78, [0.20, 1.36] | ⨁⨁⭘⭘  Low |

^a^ Most information is from studies rated as moderate or high risk of bias.

^b^ Heterogeneity in the I^2^ test >50%.

^c^ The digital health interventions is compared directly with the similar controls.

^d^ The 95% CI excludes pooled effect sizes that are not clinically significant.
